# Enhancing oligodendrocytes generation and myelin renewal by vitamin C mitigate Parkinson-relevant phenotypes in a murine model of Parkinson’s disease

**DOI:** 10.3389/fncel.2026.1761155

**Published:** 2026-02-04

**Authors:** Shihao Cui, Shengyuan Wang, Min Liu, Qian Zou, Zhiyou Cai, Jingxi Ma

**Affiliations:** 1 Department of Neurology, Chongqing General Hospital, Chongqing University, Chongqing, China; 2 Chongqing Key Laboratory of Neurodegenerative Diseases, Chongqing, China; 3 Department of Pharmacy, Chongqing General Hospital, Chongqing University, Chongqing, China; 4 Department of Neurology, People’s Hospital of Chongqing New Area, Chongqing Medical University, Chongqing, China

**Keywords:** DNA hydroxymethylation, myelin, oligodendrocyte, Parkinson’s disease, vitamin C

## Abstract

Parkinson’s disease (PD) is a major neurodegenerative disease with an increasing global prevalence. In addition to progressive dopaminergic neurons degeneration, emerging evidence implicates oligodendrocyte (OL) dysfunction and impaired myelin also contribute to PD pathogenesis. Here, we observed a significant reduction of myelin basic protein (MBP) and the number of OLs in the MPTP-induced chronic PD mouse model. Vitamin C (VC) has been reported to promote myelin regeneration in the demyelination mouse model, though its underlying mechanism remains unclear. Therefore, this study investigated the therapeutic effects of VC in the mouse model of PD by the enhancement of OPC-to-oligodendrocyte differentiation and myelin renewal. Using *in vitro* oligodendrocyte precursor cell (OPC) differentiation systems, we confirmed that VC markedly enhanced the differentiation of OPC to OL. In MPTP-induced PD mice, VC treatment not only ameliorated myelin damage but also protected dopaminergic neurons, and led to a significant improvement in PD-relevant behavioral phenotype. Mechanistically, the effects of VC are mediated through the activation of Ten-eleven translocation (TET) enzymes, which promotes DNA hydroxymethylation and subsequent expression of genes essential for OL differentiation. Taken together, these findings suggest that promoting OPC-to-oligodendrocyte differentiation and myelin repair by VC could serve as a promising therapeutic strategy in PD.

## 1 Introduction

PD is the second most common neurodegenerative disorder worldwide and its burden is rapidly escalating with population aging (Poewe et al., 2017). The main pathological changes of PD are the progressive loss of dopaminergic neurons in the substantia nigra (SN) area and abnormal aggregation of α-synuclein, leading to motor dysfunction including rest tremor, myotonia, bradykinesia and posture gait disorder (Jankovic and Tan, 2020; Morris et al., 2024). Although the precise pathogenesis of PD remains incompletely understood, growing studies indicate that multiple factors like oxidative stress, mitochondrial dysfunction, neuroinflammation, and misfolding of α-synuclein play critical roles in driving disease progression (Pickrell and Youle, 2015; Pajares et al., 2020; Vijiaratnam et al., 2021; Morris et al., 2024). Current treatment strategies for PD primarily rely on pharmacological interventions, such as dopamine replacement therapy, which can alleviate symptoms but do not restore lost neuronal function or halt disease processes (Elsworth, 2020; Vijiaratnam et al., 2021). Furthermore, long-term drug treatment is often associated with side effects. Therefore, there is an urgent need to develop novel therapeutic strategies to reverse pathological processes and overcome the limitations of existing treatments.

Emerging evidence suggests that neurodegeneration in PD extends beyond the dopaminergic system, encompassing widespread brain networks and multiple cell types, including glial populations that have been historically overlooked in therapeutic development (Smajić et al., 2022; Zhang et al., 2025). In particular, functional imaging studies have revealed that PD patients often exhibit white matter lesions (WMLs) and myelin loss, which is increasingly recognized as a significant contributor to both motor and non-motor symptoms of the disease (Bohnen and Albin, 2011; Boshkovski et al., 2022; Yang et al., 2023; Jiang et al., 2025). OLs are the myelinating glia of the central nervous system (CNS) that arise from OPCs (van Tilborg et al., 2018). Furthermore, single-cell transcriptomic analyses further reveal altered OL lineage states and dysregulated myelin genes in PD brains (Han et al., 2022; Dehestani et al., 2024; Barba-Reyes et al., 2025). This suggests that OL dysfunction and subsequent demyelination may contribute directly to axonal vulnerability and impair dopaminergic signaling.

OLs wrap axons with myelin to increase conduction velocity while supplying neurons with a plethora of metabolic and trophic factors essential for their survival (Simons and Nave, 2015; Mot et al., 2018). In the adult CNS, myelin sheaths maintain essentially unchanged. However, OPCs remain abundant and can differentiate into OLs to generate the new myelin throughout life (Hughes et al., 2018; Wang et al., 2020). Harnessing the regenerative capacity of OPCs to promote their differentiation and remyelination thus represents a promising therapeutic target for neuroprotection in PD.

VC presents a compelling candidate for such a strategy because it plays a crucial role in the development of the nervous system and its functional maintenance (Kocot et al., 2017; Lykkesfeldt and Tveden-Nyborg, 2019). Moreover, VC has been reported to promote remyelination in mouse demyelination models, but the mechanism is still unclear (Guo et al., 2018). As a cofactor for TET enzymes, VC can regulate the activity of TET-mediated DNA demethylation (Rasmussen and Helin, 2016; Gao et al., 2019), and TET enzymes have been reported to play important roles in OL formation and remyelination (Zhao et al., 2014; Moyon et al., 2021). However, despite promising preclinical evidence in other neurological contexts, the specific effects of VC on oligodendrocyte biology in PD models remain largely unexplored.

Therefore, this study investigates the hypothesis that VC administration can mitigate PD-related phenotypes by enhancing oligodendrocyte generation and myelin renewal through TET enzyme activation. Using the well-established MPTP (1-methyl-4-phenyl-1,2,3,6-tetrahydropyridine) mouse model that recapitulates key features of PD pathology, we systematically evaluated the effects of VC supplementation on behavioral deficits, dopaminergic neuronal survival, myelin integrity, and OL differentiation. In conclusion, our findings indicate the therapeutic potential of enhancing OL generation and myelination in PD-related phenotypes.

## 2 Materials and methods

### 2.1 Chronic MPTP-induced PD mouse model and pharmacological intervention

Male C57BL/6 mice (8–10 weeks) were purchased from Hunan SJA Laboratory Animal Co., Ltd. (Hunan, China) and group housed in standard mouse cages with a 12/12-h light/dark schedule and a controlled temperature (25 °C ± 2 °C). Food and water were provided ad libitum. The mice were allowed to acclimate to their housing environment for 1 week post arrival. Behavioral tests and drug treatment were performed during the light phase. All animal experiments procedures were approved by the Research Ethics Committee of Chongqing Medical University and conformed to the National Institutes of Health Guide for the Care and Use of Laboratory Animals.

To establish an animal model of PD, mice received intraperitoneal (i.p.) injections of MPTP (20 mg/kg in saline, MedChemExpress, 15608) together with probenecid (250 mg/kg in saline, MedChemExpress, B0545) twice a week for 5 weeks. Probenecid was administered 30–60 min prior to MPTP injections to retard renal clearance of toxic metabolites of MPTP. Control mice received saline (i.p.) at the same time. For drug treatment, the PD mice received daily i.p. injections of vitamin C (200 mg/kg in saline, Sigma-Aldrich, 49752) or saline for the whole experimental duration.

### 2.2 DA detection by ELISA

Mouse striatum DA levels were measured using the DA ELISA kit (Shanghai yuanju Bio, YJ002024). Briefly, mouse striatum was homogenized mechanically in normal saline under an ice water bath, followed by centrifuging at 3,000 rpm for 10 min, and then taking the supernatant to be tested. After incubation with the HRP-conjugate reagent for 60 min at 37 °C and washing with wash solutions for 5 times, the samples were reacted with TMB substrate for 15 min at 37 °C, terminated by the addition of stop solution, and finally read the Optical Density (O.D.) at 450 nm using a microtiter plate reader. The standard curve was generated according to the concentration and O.D. of the DA standard provided in the kit. DA concentrations in tissue samples were calculated by interpolation from the standard curve using a four-parameter logistic curve-fitting algorithm.

### 2.3 Behavioral testing

Rotarod Test. Motor coordination and balance were assessed using an accelerating rotarod apparatus (Jiangsu SansBio, China). Each mouse was given a training session at a constant low speed (4 rpm) for 300 s (three 5-min trials) to acclimate them to the rotarod apparatus for a continuous period of 3 days. On the test day, each mouse was placed on the rotarod with increasing speed, from 4 rpm to 40 rpm in 300 s. The latency to fall from the rod was automatically recorded. Each mice received three trials with a minimum inter-trial interval of 30 min. The average latency to fall across the three trials was calculated for each mouse and used for subsequent statistical analysis.

Pole Test. To assess motor coordination and bradykinesia, mice were put upward on the top of a rough surfaced pole (height: 50 cm, diameter:1 cm), and the time to climb down the pole was recorded. The maximum cutoff time to stop the test and recording was 30 s. Each mouse was tested three times with a minimum of 30 min resting interval in between, and the average time was used for statistical analysis. All mice were trained to perform the pole task over the three trials before the test.

Y-maze. Novel arm exploration, which is a measure of spatial working memory, was evaluated using a Y-shaped maze constructed of 3 arms (40 cm long × 8 cm wide × 15 cm high) at an angle of 120° from each other. The test consisted of two phases separated by a minimum of 1 h inter-trial interval. During the first phase, one arm (designated as the “novel arm”) was blocked with a removable partition, and mice were allowed to freely explore the two accessible arms for 5 min. Mice were then returned to their home cages. In the second phase, the partition was removed, and the mouse was placed back into the start arm with free access to all three arms for 5 min. Using ANY-maze software, we recorded the time spent in the novel arm during the second phase. Between trials, the Y maze arms were cleaned with 70% ethanol to eliminate odour and residues.

Gait Analysis. Gait assessment was conducted using a CatWalk XT automated gait analysis system (Noldus Information Technology, Wageningen, The Netherlands). Prior to testing, mice were allowed to traverse the horizontal glass walkway freely for 5–10 min to reduce anxiety and ensure natural walking behavior. On the test day, mice were positioned at one of the ends of the walkway and ran spontaneously towards the other end. For data acquisition, a minimum of three uninterrupted runs per mouse were recorded. Representative footprint maps were generated automatically by the CatWalk XT software. The major parameters including run duration and average speed were exported for statistical analysis, with the mean values from the three compliant runs used as the final measurement for each mouse. The walkway was cleaned with 70% ethanol between each trial to eliminate odour and residues.

### 2.4 Single-cell RNA sequencing

scRNA-seq was performed at Personal Biotechnology (Shanghai, China). The whole experiment included sample preparation, library construction, sequencing, and data analysis.

Sample preparation. Brain tissues were dissected from the MPTP mice and controls (*n* = 3). Tissues were cut up on ice and washed with 1 × PBS, then separated into single cells in the separation solution (37 °C water bath, 100 rpm oscillation for 20 min). The overall cell viability was confirmed by trypan blue exclusion to exceed 85% before processing.

Library construction and sequencing. Single-cell suspensions were loaded to 10x Chromium according to the manufacturer’s instructions of 10x Genomics Chromium Single-Cell 3′ kit (V3). The following cDNA amplification and library construction steps were performed according to the standard protocol. Libraries were sequenced on an Illumina NovaSeq 6000 sequencing system with 150 bp paired-end mode.

Data analysis. Raw sequencing data were processed to remove low-quality reads and aligned to the mouse genome (mm10 from 10X Genomics) using Cell Ranger (v.7.1.0). The resulting gene expression matrices merged together using Seurat package v5. Cells that have >20% mitochondrial counts were filtered to remove low quality cells. Cells with fewer than 400 genes (empty droplets) or more than 7,500 genes (apparent doublets) were also filtered out. Downstream data analysis, including normalization, dimensionality reduction, and clustering, were performed by the Seurat software package. The Gene Ontology (GO) and Kyoto Encyclopedia of Genes and Genomes (KEGG) analysis were used with the clusterProfiler R package. Pathways with p_adj value less than 0.05 were considered as significantly enriched.

### 2.5 Western blots

Mice were anesthetized and perfused with saline. Brain tissues were separated and homogenized on ice with RIPA lysis buffer (Beyotime, P0013B) supplemented with a protease and phosphatase inhibitor cocktail (Beyotime, P1045). Then, the homogenates were centrifuged at 12,000 × g for 20 min at 4 °C, and supernatants were collected. The protein concentration was determined by BCA Assay Kit (Beyotime, P0010S). Equal amounts of protein were mixed with sample loading buffer (Biosharp, BL502A) and heated for 5 min at 100 °C. The protein samples were then separated on 12.5% sodium dodecyl sulfate-polyacrylamide gel electrophoresis (SDS-PAGE) gels and subsequently transferred to polyvinylidene difluoride (PVDF) membranes (Merck Millipore, ISEQ00010). Following a standard protocol, the PVDF membrane were blocked in 5% nonfat milk in Tris-buffered saline containing 0.1% Tween-20 (TBST) for 1 h at room temperature. Then the membranes were incubated overnight at 4 °C with rat anti-MBP antibody (Abcam, ab7349, 1:2,000), rabbit anti-TH antibody (Proteintech, 25859-1-AP, 1:2,000), or rabbit anti-GAPDH antibody (Proteintech, 10494-1-AP, 1:5,000). After washing, the membranes were incubated for 1 h at room temperature with the corresponding horseradish peroxidase-labeled (HRP) secondary antibody: goat anti-rabbit IgG (Beyotime, A0208, 1:2,000) or goat anti-rat IgG (Beyotime, A0192, 1:2,000). The protein signals were detected by enhanced chemiluminescence (ECL) detection reagent (Shanghai epizyme, SQ203) and imaged on the Fusion imaging system (Vilber, China). The intensity of each protein band was quantified with ImageJ and normalized by GAPDH.

### 2.6 Cell culture medium

DMEM20S: DMEM (Gibco, 11960), 4 mM L-glutamine (Sigma, G8540), 1 mM sodium pyruvate (Sigma, P2256), 20% FBS (Sigma, F8318), 50 U/ ml penicillin and 50 μg/mL streptomycin (Gibco, 15140). OPC medium: DMEM/F12 (Sigma, D8437), 2% B27 Supplement (Invitrogen, 17504044), 10 ng/mL bFGF (Peprotech, 100-18B), 10 ng/mL PDGF-AA (Peprotech, 100-13A), 100 U/mL penicillin and 100 μg/mL streptomycin (Gibco, 15140). Differentiation medium: DMEM/F12 (Sigma, D8437), 2% B27 Supplement (Invitrogen, 17504044), VC, 100 U/mL penicillin and 100 μg/mL streptomycin (Gibco, 15140).

### 2.7 Primary rat OPC culture and differentiation

To obtain primary OPCs, the brains were dissected from neonatal SD rat (P1-2). Then, the cerebral cortices were collected after the meninges were removed in ice-cold Hanks’ balanced salt solution (HBSS). The cortices tissues were manually chopped into small pieces and digested in HBSS containing 0.25% trypsin (Sigma, T1426) and 0.2 mg/mL DNase I (Roche, 10104159001) for 15 min at 37 °C. The digestion was terminated by the addition of DMEM20S. The tissue suspension was blown into a nearly homogenous with a glass pipette. Then, the tissue suspension was passed through a 70 μm filter, and the cells were collected by centrifuging at 1000 rpm for 5 min. Thereafter, the cells were plated into a T75 poly-D-lysine (100 μg/mL; Sigma, P0899) coated flask and cultured in DMEM20S with complete medium change every 3 days. After 9 days, the flask was shaken on a shaker at 200 rpm for 2 h at 37 °C to remove microglial cells. Then, the fresh DMEM20S was added, and the flask was shaken at 200 rpm for 18 to 20 h. The cell suspension was collected and transferred to an untreated petri dish. After incubating petri dishes for 45 min in tissue culture incubator, the culture medium was centrifuged at 1000 rpm for 5 min to collect OPCs. OPCs were seeded onto poly-ornithine (25 μg/mL; Sigma, P0421) plus laminin (1 μg/mL; Sigma, L2020)-coated plates and cultured in the OPC medium. Two days later, primary rat OPCs were stimulated with VC or control to generate MBP^+^ OLs for another 4 days in the differentiation medium.

### 2.8 MO3.13 human oligodendroglia cell line culture and differentiation

The MO3.13 cell line (IMMOCELL, IM-H672, Xiamen, China) was cultured in high-glucose Dulbecco’s Modified Eagle’s Medium (DMEM) (Sigma, D6429) supplemented with 20% fetal bovine serum (Sigma, F8318) and 50 U/ ml penicillin/50 μg/mL streptomycin (Gibco, 15140). For differentiation, cells were seeded onto a poly-D-lysine (100 μg/mL; Sigma, P0899) coated 24-well plates and cultured in the serum-free differentiation medium containing 150 μM VC for 4 days. Following 4 days of differentiation, MO3.13 cells were exposed to 500 μM 1-methyl-4-phenylpyridinium (MPP^+^) (AbMole, M10041) to establish PD cell model. For drug treatment, cells were incubated with MPP^+^ alone or MPP^+^ combined with VC for 48 h. Control cells received an equivalent volume of PBS.

### 2.9 Cell viability assay

Cell viability was assessed using the CellTiter-Glo® Luminescent Cell Viability Assay (Promega, G7571). MO3.13 cells were seeded into 96-well plates at a density of 4 × 10^3^ cells per well and cultured for 24 h. Subsequently, cells were treated with MPP^+^ in the presence or absence of VC for an additional 24 h. Control wells received an equivalent volume of PBS. Following treatment, plates were equilibrated to room temperature for approximately 10 min. One hundred microliters of CellTiter-Glo® Reagent was added to the cell culture medium in each well. The plates were placed on an orbital shaker for 2 min to induce cell lysis, followed by a 10-min incubation at room temperature to stabilize the luminescent signal. Luminescence was recorded using a microplate reader. Results were expressed as the percentage of cell viability relative to untreated control cells.

### 2.10 Immunofluorescence assay

For cell staining, cells were fixed with 4% PFA for 15 min at room temperature and blocked in blocking buffer (Beyotime, P0260) for 30 min at room temperature. Then cells were incubated with antibody to MBP (Abcam, ab7349, 1:500), NG2 (Millipore, AB5320, 1:200), Tuj1 (Merck, MAB1637, 1:200), Iba1 (Cell Signaling Technology, 17198 T, 1:200) or GFAP (Abcam, ab7260, 1:500) at 4 °C overnight. After washing with PBS, the cells were stained with secondary antibody conjugated to Alexa Fluor 488 or Alexa Fluor 555 (1:1,000, Thermo Fisher) for 1 h at room temperature. The nuclei were counterstained with DAPI (Beyotime, C1005) at room temperature. High-content images were then acquired and quantified using Operetta (PerkinElmer).

For analysis of MBP or TH intensity in mouse brain tissues, the mouse brain was made into a frozen section. The brain slices were blocked and permeated in blocking buffer (Beyotime, P0260) for 1 h at room temperature, then incubated with anti-MBP antibody (Abcam, ab7349, 1:500), anti-GST-pi antibody (MBL Corp, MBL312, 1:500) or anti-TH antibody (Proteintech, 25859-1-AP, 1:500) at 4 °C overnight. After thoroughly washing, slices were stained with secondary antibody conjugated to Alexa Fluor 488 or Alexa Fluor 555 (1:1,000, Thermo Fisher) for 1 h at room temperature, and nuclei were stained with DAPI (Beyotime, C1005). Fluorescent images were captured using a Leica DMi8 Thunder Imager fluorescent microscope and analyzed blindly by Image-Pro Plus.

For DNA 5hmC staining, fixed cells were first permeabilized, denatured with 2 N HCl for 30 min, and neutralized with 100 mM Tris–HCl (pH 8.0) for 5 min at room temperature. Then, cells were blocked in blocking buffer (Beyotime, P0260) for 30 min at room temperature and then sequentially incubated with anti-5hmC antibody (Active Motif, #39769, 1:1,000) at 4 °C overnight and secondary antibody conjugated to Alexa Fluor 488 (1:1,000, Thermo Fisher) for 1 h at room temperature. The nuclei of cells were stained with DAPI (Beyotime, C1005). Fluorescent images were captured using a Leica DMi8 Thunder Imager fluorescent microscope and analyzed blindly by Image-Pro Plus.

### 2.11 RNA-sequencing

RNA-seq were performed by BGI-Shenzhen, China. Briefly, total RNA was extracted from the brain tissues using Trizol (Invitrogen, Carlsbad, CA, USA) according to manual instruction. The RNA library construction was performed using Optimal Dual-mode mRNA library Prep Kit (BGI-Shenzhen, China) and sequenced on G400 platform (BGI-Shenzhen, China) with single end 50 bases reads. The sequencing data was filtered with SOAPnuke (v1.5.6) and the clean reads were mapped to the mouse reference genome using HISAT2 (v2.1.0). RSEM (v1.3.1) was used to calculate the expression levels of gene and transcript abundance was quantified as Transcripts Per Million (TPM).

### 2.12 Statistical analysis

Data were analyzed with GraphPad Prism software (V 8.0.0). For comparison between two groups, statistical evaluation was carried with two-tailed Student’s *t*-test. For analyses involving multiple groups, one-way ANOVA followed by Tukey’s test was performed. For all statistical tests, *p* values < 0.05 were considered statistically significant. All error bars show the standard error of the mean (SEM).

## 3 Results

### 3.1 OL and myelin loss in the brain of MPTP-induced PD mouse model

**Figure 1:**
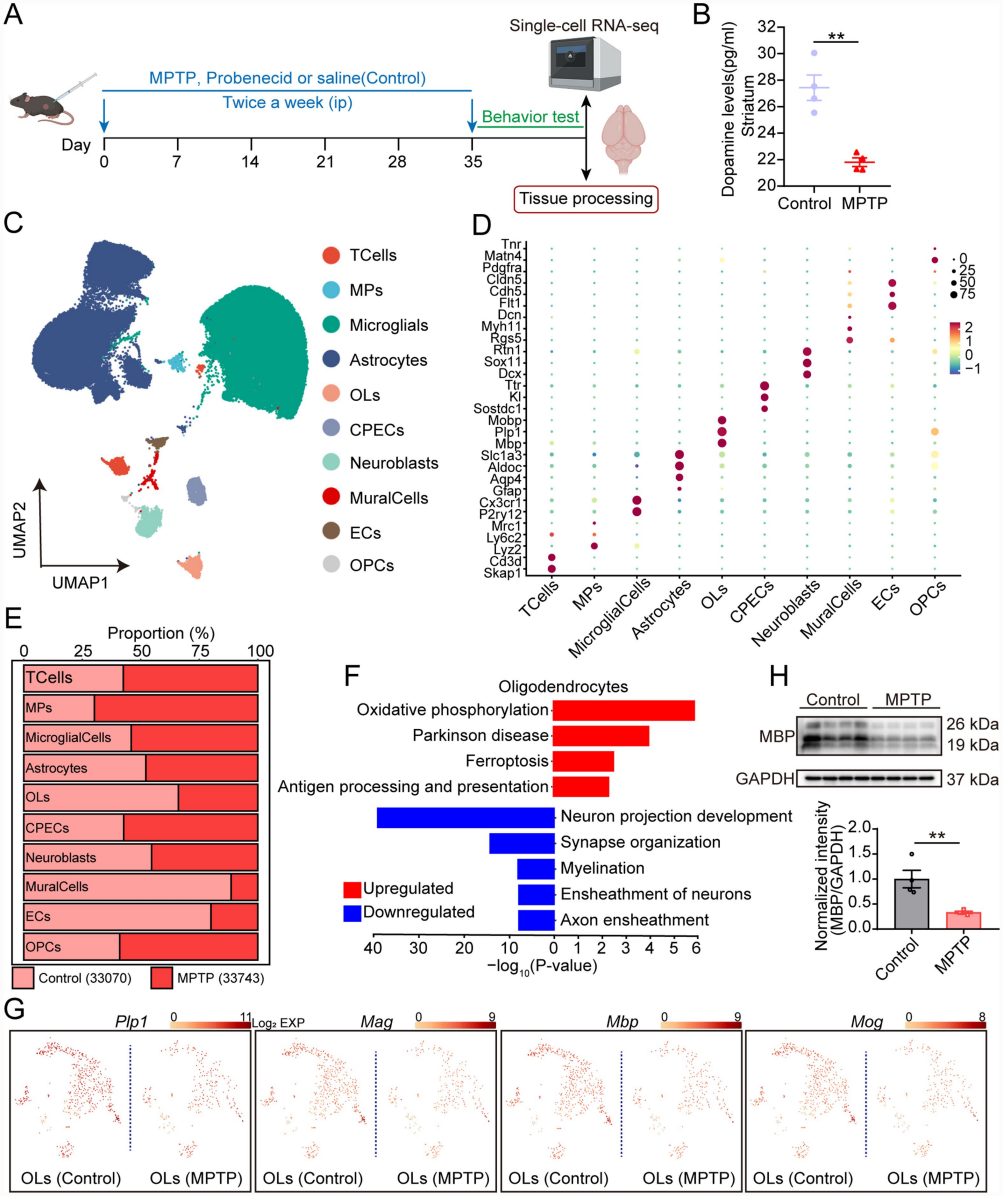
OL and myelin loss in the brain of MPTP-induced PD mouse model. **(A)** Schematic illustrating *in vivo* experiments schedule. Mice received twice-weekly intraperitoneal injections of MPTP (20 mg/kg) together with probenecid (250 mg/kg) or saline (control) for 5 weeks, followed by tissue processing and single-cell RNA sequencing. **(B)** Quantification of dopamine (DA) levels in the striatum of control and MPTP mice by ELISA. Data are presented as mean ± SEM (*n* = 4), **p < 0.01 versus control group (Student’s *t*-test). **(C)** UMAP plots demonstrating the cell distribution from six mouse brain tissues (*n* = 3 for control group; *n* = 3 for MPTP group), colored by annotated cell types. **(D)** Dot plot showing the expression of canonical marker genes across identified cell cluster. Dot size reflects the proportion of subtype cells expressing a specific gene, while color intensity denotes the average expression level. **(E)** Bar plot indicating the proportions of annotated cell types in control and MPTP mouse brains. **(F)** Functional enrichment analysis of differentially expressed genes showing enriched down-regulated (blue) and up-regulated (red) pathways in OLs from MPTP group compared to control group. Significantly enriched GO biological process terms and KEGG pathways are shown. **(G)** Feature plots presenting the expression levels of myelin-associated genes (Plp1, Mag, Mbp, Mog) in OLs from control and MPTP mice. The color bar indicates the log_2_ transformed of the gene expression. Color intensity ranging from yellow to red indicates expression levels from low to high. **(H)** Western blot image and statistical analysis of MBP in brain tissues from control and MPTP mice. GAPDH was used as a loading control. Data are presented as mean ± SEM (*n* = 4), **p < 0.01 versus control group (Student’s *t* test).

To investigate myelin alterations in PD, we established a chronic PD mouse model through intraperitoneal injection of MPTP twice weekly for 5 weeks(Ayerra et al., 2024) (Figure 1A). Following MPTP toxin injection, dopamine (DA) levels in the striatum region of brain were significantly reduced in MPTP-treated mice compared to controls (Figure 1B), consistent with the characteristic dopaminergic neurodegeneration observed in PD. To explore the change of oligodendrocytes in PD, we further performed scRNA-seq on brains from both control and MPTP-induced PD model mice. We obtained a transcriptome dataset comprising 66,813 cells after excluding low-quality cells. Of these, 33,070 cells were from control group, and 33,743 cells were from MPTP group. Uniform manifold approximation and projection (UMAP) visualization results identified 10 distinct cell populations based on canonical marker genes, including T cells, macrophages (MPs), microglia, astrocytes, OLs, choroid plexus epithelial cells (CPECs), neuroblasts, mural cells, endothelial cells (ECs), and OPCs (Figures 1C,D). Notably, analysis of cell population proportion revealed a significant decrease in the number of oligodendrocytes in the PD group relative to control (Figure 1E). Furthermore, GO and KEGG functional enrichment analysis revealed a significant downregulation of myelination, ensheathment of neurons, and axon ensheathment in PD OLs, whereas oxidative phosphorylation, PD, and ferroptosis were upregulated (Figure 1F). Consistently, gene expression analysis within the oligodendrocyte cluster showed substantial decreases in key myelin-associated genes including *Plp1*, *Mbp*, *Mog*, and *Mag* in PD group relative to control (Figure 1G). These data indicate that MPTP exposure induces a state of functional impairment in oligodendrocytes. Such functional deficits can contribute to progressive demyelination and loss of oligodendrocyte-mediated metabolic support to axons, which represents a critical component of oligodendrocyte pathology in PD. To further evaluated myelin damage, we performed Western blot analysis, which demonstrated a significant reduction of MBP in PD mouse brains compared to controls (Figure 1H). Collectively, these findings demonstrate the presence of demyelinating pathology in the MPTP-induced chronic PD mouse model, characterized by OL loss and decreased myelin protein expression.

### 3.2 VC enhances OPC-to-oligodendrocyte differentiation *in vitro*

**Figure 2:**
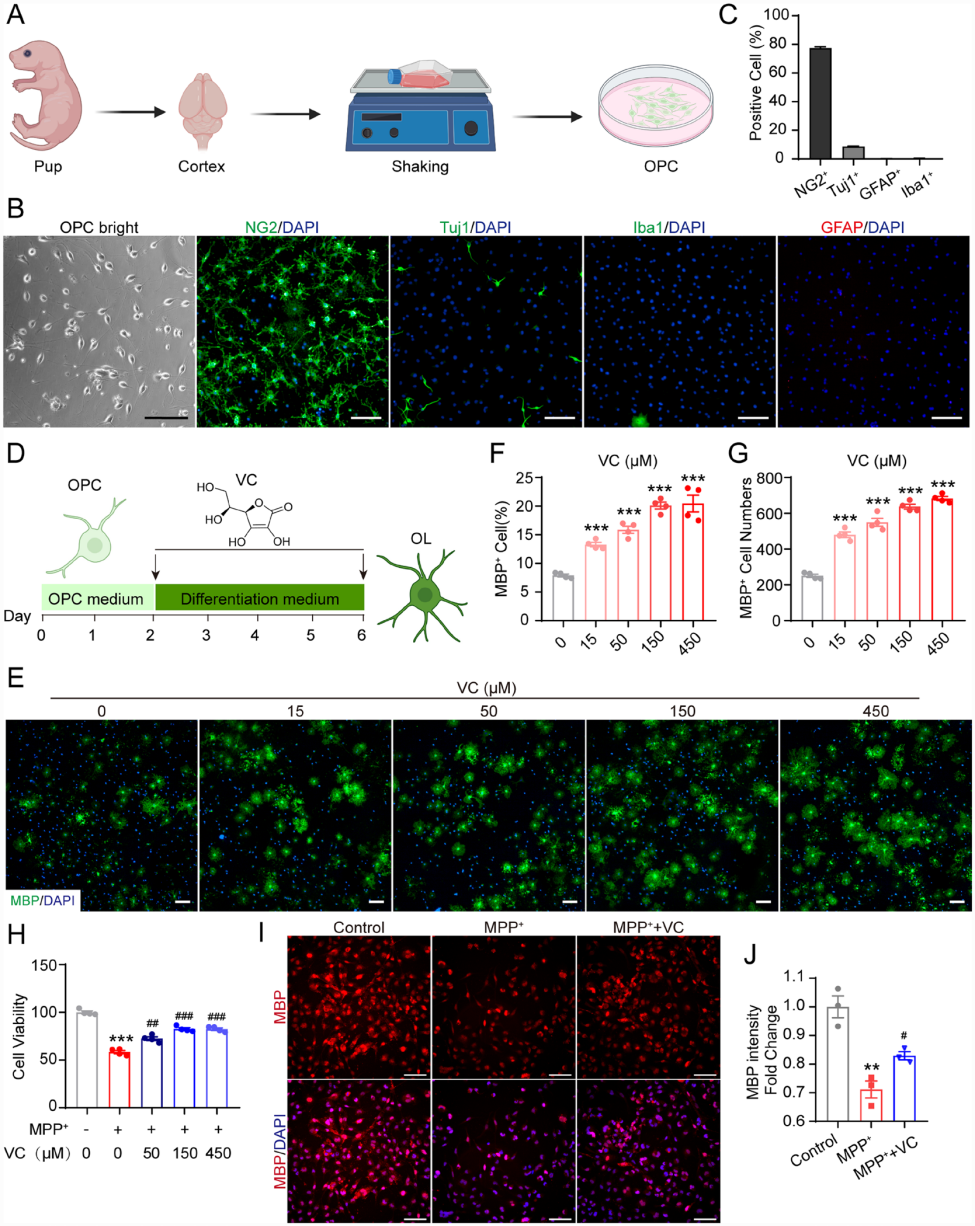
VC promotes rat primary OPC to OL differentiation. **(A)** The diagram of rat primary OPC isolation and culture *in vitro*. Mixed glial cells were mechanically dissociated from P1-2 postnatal rat cortices and cultured *in vitro* for 10 days. At 10 days after plating, OPCs were enriched by shaking and plated for differentiation assays. Created in BioRender. **(B)** Representative bright-field image of isolated OPCs and immunofluorescent staining of specific markers of OPC (NG2), neuron (Tuj1), microglial (Iba1), and astrocyte (GFAP). The nuclei were labeled with DAPI. Scale bars, 100 μm. **(C)** Percentage of NG2^+^ cells, Tuj1^+^ cells, Iba1^+^ cells, and GFAP^+^ cells among DAPI^+^ cells in **(B)**. Data are presented as mean ± SEM (*n* = 3). **(D)** Experimental design for *in vitro* OPC differentiation. Primary OPCs were cultured in OPC medium for 2 days and then switched to differentiation medium containing various concentrations of VC for 4 days to induce maturation into OLs. **(E)** Representative images of MBP^+^ OLs induced from primary OPCs with various concentrations of VC for 4 days. The nuclei were labeled with DAPI. Scale bars, 100 μm. **(F,G)** Percentage **(F)** and number **(G)** of MBP^+^ OLs in **(E)**. Data are presented as mean ± SEM (*n* = 4), ***p < 0.001 versus control group (0 M VC) (Student’s *t* test). **(H)** Viability of MO3.13 cells treated with MPP^+^(500 M) in combination with various concentrations of VC for 24 h. Data are presented as mean ± SEM (*n* = 4), ***p < 0.001 versus control group (0 μM MPP^+^, 0 M VC), ^##^*p* < 0.01, ^###^*p* < 0.001 versus MPP^+^ group (500 M MPP^+^, 0 M VC) (Student’s *t* test). **(I,J)** immunofluorescent staining and statistical evaluation of MBP expression in MO3.13 cells. Scale bars, 100 m. Data are presented as mean ± SEM (*n* = 3), **p < 0.01 versus control group, ^#^*p* < 0.05 versus MPP^+^ group (Student’s *t* test).

We hypothesized that these white matter alterations might contribute to PD-related functional impairments. This led us to consider whether promoting remyelination by small-molecule drug could ameliorate these deficits. VC has been previously reported to promote myelin regeneration in cuprizone-induced demyelination model by enhancing OPC-to-oligodendrocyte differentiation and maturation (Guo et al., 2018). Therefore, to investigate whether VC could enhance OPC-to-oligodendrocyte differentiation as a potential therapeutic strategy for PD, we first established a rat primary OPC differentiation system *in vitro* to evaluate the effect of VC on oligodendrocytes formation. Briefly, primary OPCs were isolated from P1-2 postnatal rat cortices by a shaking method based on differential adherent properties of glia (Chen et al., 2007) (Figure 2A). OPCs were cultured in OPC medium for 2 days, which exhibited the characteristic bipolar or multipolar morphology (Figure 2B). We further assessed the purity of the culture by immunofluorescent staining (Figures 2B,C). The majority of isolated cells were positive for NG2, a canonical marker of OPCs, with only a small fraction of neurons labeled by Tuj1 and negligible presence of GFAP-positive astrocytes or Iba1-positive microglia. These results indicate that our isolation method successfully generated high-purity primary OPCs suitable for subsequent differentiation assays. Following a 2-day proliferation period in OPC medium containing bFGF and PDGF-AA, these OPCs were further differentiated into MBP^+^ mature OLs in the differentiation medium lacking growth factor with varying concentrations of VC for 4 days (Figure 2D). As expected, VC dose-dependently increased the percentage and the number of MBP^+^ cells and reached a plateau at a concentration of 150 μM (Figures 2E–G). These data confirm that VC promotes the differentiation of primary rat OPCs into mature OLs *in vitro*.

To determine whether PD-related toxins can directly affect OL lineage cells, we performed in vitro experiments using the human oligodendroglial cell line MO3.13. MO3.13 cells were treated with MPP^+^, a neurotoxin that recapitulates key PD-associated cellular damage. Cell viability assay results demonstrated that MPP^+^ treatment significantly reduced MO3.13 cell viability, confirming direct cytotoxicity toward oligodendrocytes (Figure 2H). Importantly, co-treatment with VC partially rescued cell viability (Figure 2H). Furthermore, immunofluorescence staining for MBP revealed that MPP^+^ exposure markedly decreased MBP expression in MO3.13 cells (Figures 2I,J). VC co-treatment significantly attenuated this reduction (Figures 2I,J), suggesting that VC not only protects oligodendrocyte survival but also preserves their myelinating capacity. These findings confirm that MPP^+^ exerts direct toxicity on oligodendrocyte lineage cells, supporting the view that OLs dysfunction in the MPTP mouse model involves primary oligodendroglial pathology, and that VC can act directly on OLs to counteract this damage.

### 3.3 VC improves PD-relevant behavioral phenotypes in MPTP-induced PD mouse model

**Figure 3:**
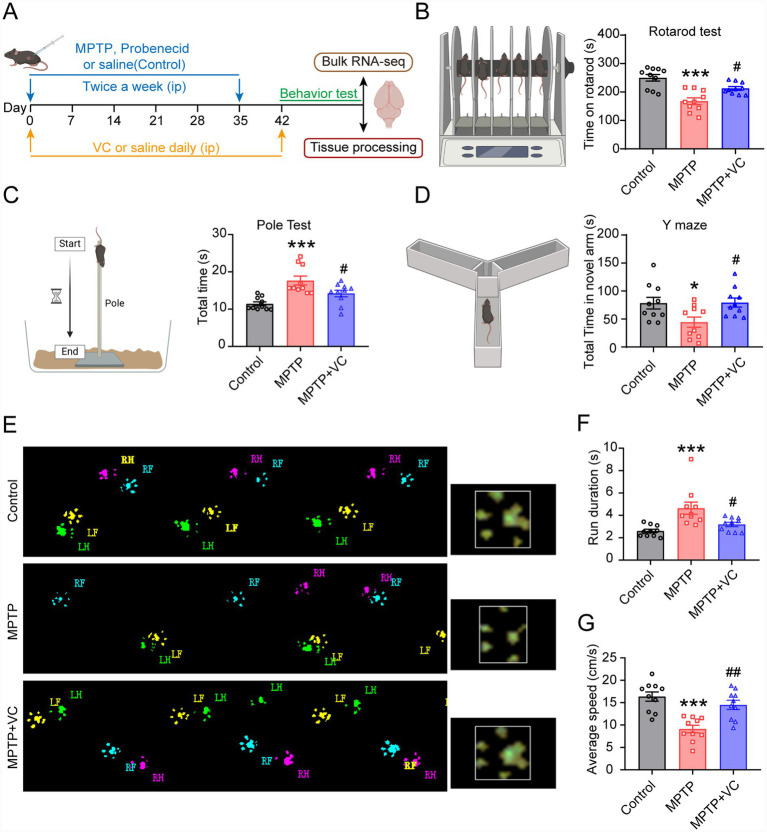
VC improves PD-relevant behavioral phenotypes in MPTP-induced PD mouse model. **(A)** Schematic illustrating *in vivo* experiments schedule. MPTP mice received daily intraperitoneal injections of VC (200 mg/kg) or saline for 6 weeks, followed by behavioral assessments, tissue processing, and bulk RNA sequencing. **(B)** Quantification of the time spent on the rotating rod in the rotarod test **(C)** Quantification of the total time taken to descend in the pole test. **(D)** Quantification of the total time exploring the novel arm in the Y-maze test. **(E)** Representative footprint images from the CatWalk XT gait analysis system, illustrating the paw print patterns of control, MPTP + saline (MPTP), and MPTP + VC mice (RH, right hind; RF, right front; LH, left hind; LF, left front). **(F,G)** Quantification of the run duration **(F)** and the average speed **(G)** in the control, MPTP + saline (MPTP), and MPTP + VC mice measured by the CatWalk XT gait analysis system. *n* = 10 mice in each group. All data are presented as mean ± SEM, *p < 0.05, **p < 0.01, ***p < 0.001 versus control group, ^#^*p* < 0.05, ^##^*p* < 0.01 versus MPTP group (one-way ANOVA followed by Tukey’s test).

Given the demonstrated capacity of VC to promote the generation of mature OLs in vitro, we wondered whether VC has beneficial effects on PD-related behavioral deficits. VC (200 mg/kg) or saline was administered by intraperitoneal injection once a day throughout the whole experimental period, and the behavioral phenotypes and pathology were then assessed (Figure 3A). In the rotarod test, mice in MPTP group spent significantly less time on the rod compared with controls, and VC treatment prolonged the latency on the rod (Figure 3B). In the pole test, which evaluates motor coordination and bradykinesia, the MPTP group exhibited significantly prolonged total time to reach the bottom compared with the control group, and VC treatment led to a significant improvement (Figure 3C). In the Y-maze test, MPTP mice spent less time exploring the novel arm compared to the control group, whereas VC treatment significantly increased novel arm exploration time, suggesting a potential improvement in cognitive function (Figure 3D). Gait disturbance is a major motor manifestation of PD. Thus, we further tested the gait phenotype of these mice using the CatWalk XT gait analysis system. Representative footprints showed that control mice displayed regular alternating footfall patterns with uniform stride lengths and consistent step counts (Figure 3E). In contrast, the footfall patterns of MPTP mice exhibited irregular gait sequences and variable stride lengths (Figure 3E). VC treatment significantly improved these gait deficits (Figure 3E). In addition, motor functions detected by two specific parameters (namely, time to traverse a defined distance and average velocity) were significantly rescued by VC treatment (Figures 3F,G). Collectively, these behavioral data demonstrate that VC administration effectively mitigates both motor and cognitive impairments in the MPTP-induced mouse model of PD.

### 3.4 VC ameliorates myelin damage and protects dopaminergic neurons in MPTP-induced PD mouse model

**Figure 4:**
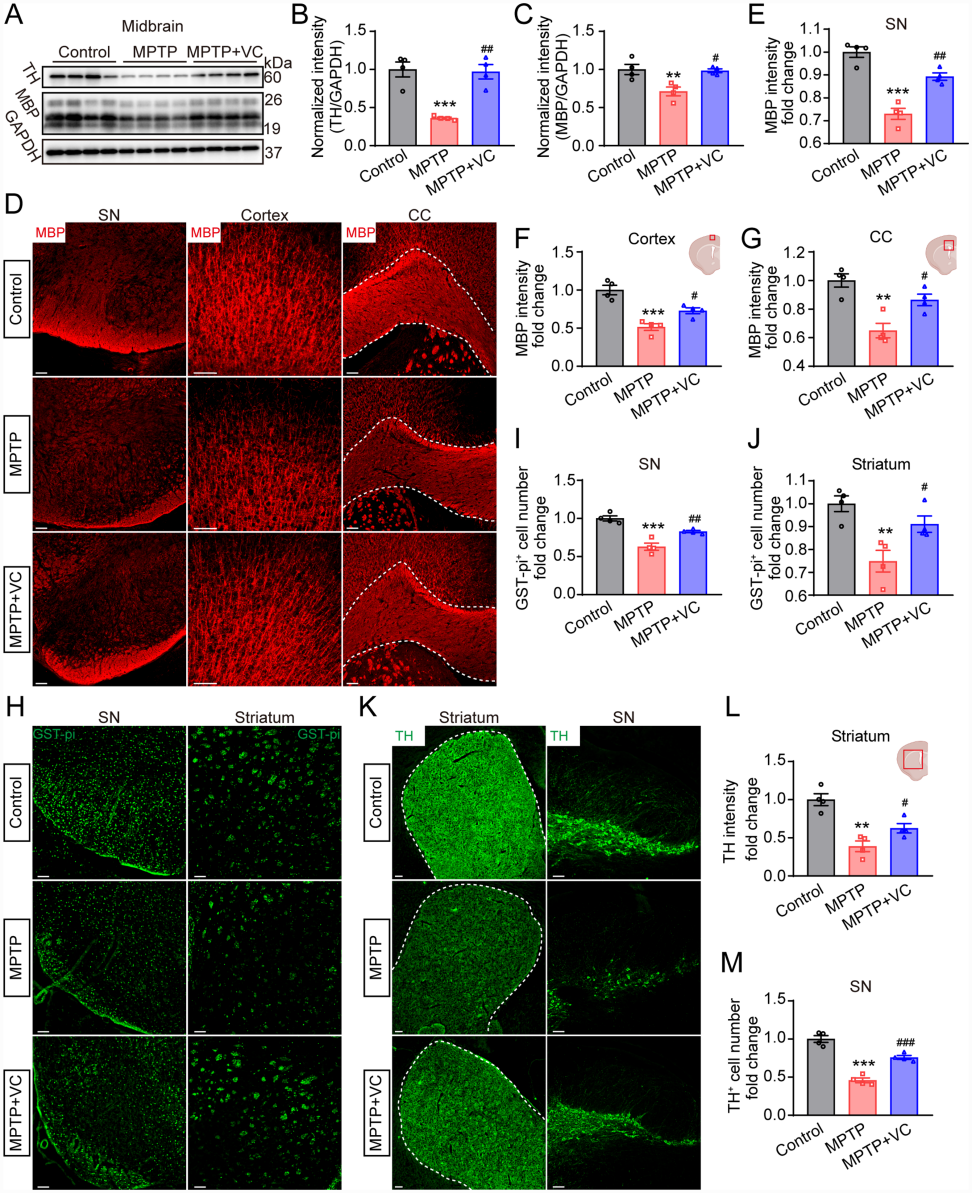
VC ameliorates myelin damage and protects dopaminergic neurons in MPTP-induced PD mouse model. **(A–C)** Western blot images **(A)** and statistical analysis of TH **(B)** and MBP **(C)** in the midbrain tissues from control, MPTP, and MPTP+VC mice. GAPDH was used as a loading control. **(D)** Immunostaining of MBP in SN, cortex, and corpus callosum of control, MPTP, and MPTP+VC mice. Scale bars, 100 μm. **(E–G)** Statistical analysis of the fluorescence intensity fold change of MBP in **(D)**. **(H)** Immunostaining of GST-pi in SN and striatum of control, MPTP, and MPTP+VC mice. Scale bars, 100 μm. **(I,J)** Statistical analysis of the number of GST-pi^+^ cells in **(H)**. **(K)** Immunostaining of TH in SN and striatum of control, MPTP, and MPTP+VC mice. Scale bars, 100 μm. **(L)** Statistical analysis of the fluorescence intensity fold change of TH in **(K)**. **(M)** Statistical analysis of the number of TH^+^ cells in **(K)**. *n* = 4 mice in each group. All data are presented as mean ± SEM, *p < 0.05, **p < 0.01, ***p < 0.001 versus control group, ^#^*p* < 0.05, ^##^*p* < 0.01, ^###^*p* < 0.001 versus MPTP group (one-way ANOVA followed by Tukey’s test).

Having demonstrated that VC improves behavioral deficits in PD mice, we next investigated the underlying neuropathological changes. Loss of dopaminergic neurons in the substantia nigra midbrain is the pathological hallmark of PD. Therefore, we first examined tyrosine hydroxylase (TH) and MBP expression in the midbrain by Western blot. The results showed that the MPTP group exhibited a significant reduction in TH compared to the control group, indicative of dopaminergic neuron loss (Figures 4A,B). The loss was significantly attenuated following VC treatment (Figures 4A,B). Consistent with our earlier data (Figure 1H), the expression of MBP was also significantly diminished in the MPTP group, and this myelin damage was markedly ameliorated by VC intervention (Figures 4A,C). To further assess the potential therapeutic effects of VC regarding myelin degeneration and dopamine neuron damage in PD mice, we performed immunostaining of MBP and TH across both gray and white matter regions. In the MPTP group, MBP staining was significantly decreased in the cortex and corpus callosum compared with controls, suggesting widespread myelin damage in regions that are critical for motor and cognitive functions (Figures 4D,F,G). As expected, VC treatment led to a significant restoration of myelin in these areas (Figures 4D,F,G), which is consistent with the improvement in Y-maze performance. Myelin loss and diminished myelin renewal have been increasingly recognized as contributors to cognitive impairment, whereas enhancing myelination rescued spatial memory in aged mice (Wang et al., 2020; Chen et al., 2021). By promoting OPC-to-oligodendrocyte differentiation and myelin renewal, VC treatment likely restores white matter integrity and enhances the functional connectivity of neural circuits involved in spatial working memory, thereby improving cognitive performance in the Y-maze task. Concurrently, we quantified the mean fluorescence intensity of MBP in the SN (Figures 4D,E). We observed a marked reduction in MBP intensity in the SN of MPTP-treated mice, indicating local demyelination or myelin loss in the nigral region. VC treatment significantly restored MBP intensity, suggesting that VC-mediated protection of myelin occurs spatially adjacent to the nigrostriatal pathway.

We also performed immunofluorescence staining for GST-pi, a well-established marker for oligodendrocytes, and quantified oligodendrocyte numbers in the SN and striatum (Figures 4H–J). The results demonstrated that MPTP treatment significantly reduced the number of GST-pi^+^ oligodendrocytes in both the SN and striatum compared to the control group. VC treatment substantially rescued oligodendrocyte numbers in both regions.

Furthermore, the fluorescence intensity of TH in the striatum, which reflects dopaminergic nerve terminals, was markedly diminished in MPTP mice relative to the controls (Figures 4K,L). The administration of VC significantly reversed the loss of dopaminergic nerve terminals (Figures 4K,L). Similarly, Our results also confirm a significant loss of TH^+^ neurons in the MPTP group compared to controls (Figures 4K,M). Importantly, VC treatment significantly attenuated this loss, preserving the dopaminergic population (Figures 4K,M). Collectively, these findings indicate that VC concurrently ameliorates myelin damage and preserves dopaminergic neurons in the PD model, suggesting that myelin restoration may contribute to the attenuation of dopaminergic neurodegeneration and the consequent improvement in motor and cognitive functions.

### 3.5 TETs-mediated DNA hydroxymethylation contributes to VC-induced generation of OLs

**Figure 5:**
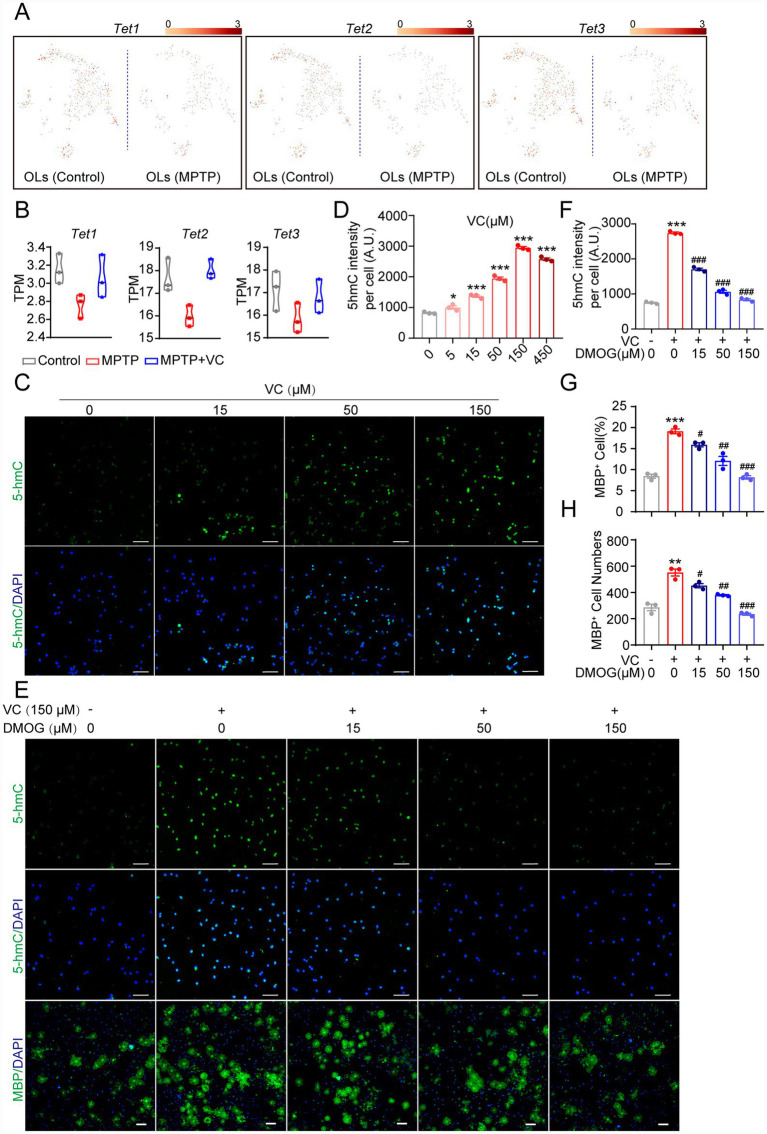
TETs-mediated DNA hydroxymethylation contributes to VC-induced generation of OLs. **(A)** Feature plots presenting the expression levels of Tet1, Tet2, and Tet3 in OLs from control and MPTP mice. The color bar indicates the log_2_ transformed of the gene expression. Color intensity ranging from yellow to red indicates expression levels from low to high. **(B)** Violin plots displaying the expression levels (TPM, transcripts per million) of Tet1, Tet2, and Tet3 in brain tissues from control, MPTP, and MPTP+VC mice. *n* = 3 mice in each group. **(C)** Immunostaining of 5-hmC in OLs induced from primary OPCs with various concentrations of VC for 4 days. The nuclei were labeled with DAPI. Scale bars, 50 μm. **(D)** Statistical analysis of the fluorescence intensity of 5-hmC in **(C)**. Data are presented as mean ± SEM (*n* = 3), *p < 0.05, ***p < 0.001 versus control group (0 M VC) (Student’s *t* test). **(E)** Immunostaining of 5-hmC or MBP in OLs induced with VC (150 M) in combination with various concentrations of DMOG for 4 days. The nuclei were labeled with DAPI. Scale bars, top panels (5-hmC), 50 m; bottom panels (MBP), 100 m. **(F-H)** Statistical analysis of the fluorescence intensity of 5-hmC **(F)** and the percentage **(G)** and number **(H)** of MBP^+^ OLs in **(E)**. Data are presented as mean ± SEM (*n* = 3), **p < 0.01, ***p < 0.001 versus control group (0 μM VC, 0 M DMOG), ^#^*p* < 0.05, ^##^*p* < 0.01, ^###^*p* < 0.001 versus VC group (150M VC, 0M DMOG) (Student’s *t* test).

VC has been identified as an essential coactivator for TET dioxygenases, which catalyze the conversion of 5-methylcytosine (5mC) to 5-hydroxymethylcytosine (5hmC) in DNA, a critical step to promote DNA demethylation and favor gene expression (Wu and Zhang, 2011; Minor et al., 2013; Yin et al., 2013; Wu and Zhang, 2017). Moreover, TET enzymes have been reported to play key roles in OL formation, myelination, and remyelination by regulating the expression of genes important for OL differentiation and myelin repair (Zhao et al., 2014; Moyon et al., 2021; Zhang et al., 2021). Therefore, we speculated whether VC exerted its therapeutic effects in the PD model through TET activation. Analysis of scRNA-seq data showed that all three TET isoforms (*Tet1*, *Tet2*, and *Tet*3) were down-regulated in oligodendrocytes from MPTP mice compared to controls (Figure 5A), which may be attributable to the overall decrease in oligodendrocyte numbers. Furthermore, our bulk RNA-seq analysis indicated that while there was a trend toward reduced Tet expression in the MPTP group and a slight recovery in the VC-treated group, these changes did not reach statistical significance (Figure 5B). Next, we detected the fluorescence intensity of the DNA hydroxymethylation mark 5hmc in OLs induced from primary OPCs with various concentrations of VC. As expected, VC also dose-dependently increased the intensity of 5hmc, with the maximal effect observed at 150 μM (Figures 5C,D). This result was consistent with the aforementioned concentrations of VC for promoting the OL differentiation. Taken together, these findings suggests that the robust increase in 5hmC levels and improved oligodendrocyte differentiation observed following VC treatment are likely driven by the cofactor-mediated enhancement of TET enzymatic activity, rather than a transcriptional upregulation of Tet genes. DMOG, a non-selective TET dioxygenase inhibitor, abolished the VC-induced elevation of 5hmC in a dose-dependent manner (Figures 5E,F). Furthermore, inhibiting TET activity with DMOG also abrogated the pro-differentiation effects of VC, significantly reducing both the percentage (Figures 5E,G) and absolute number of MBP^+^ mature oligodendrocytes (Figures 5E,H). Collectively, these findings demonstrate that VC enhances OPC-to-oligodendrocyte differentiation through TET-mediated DNA hydroxymethylation, providing a possible molecular mechanism for its therapeutic efficacy in alleviating myelin damage in the PD model.

**Figure 6:**
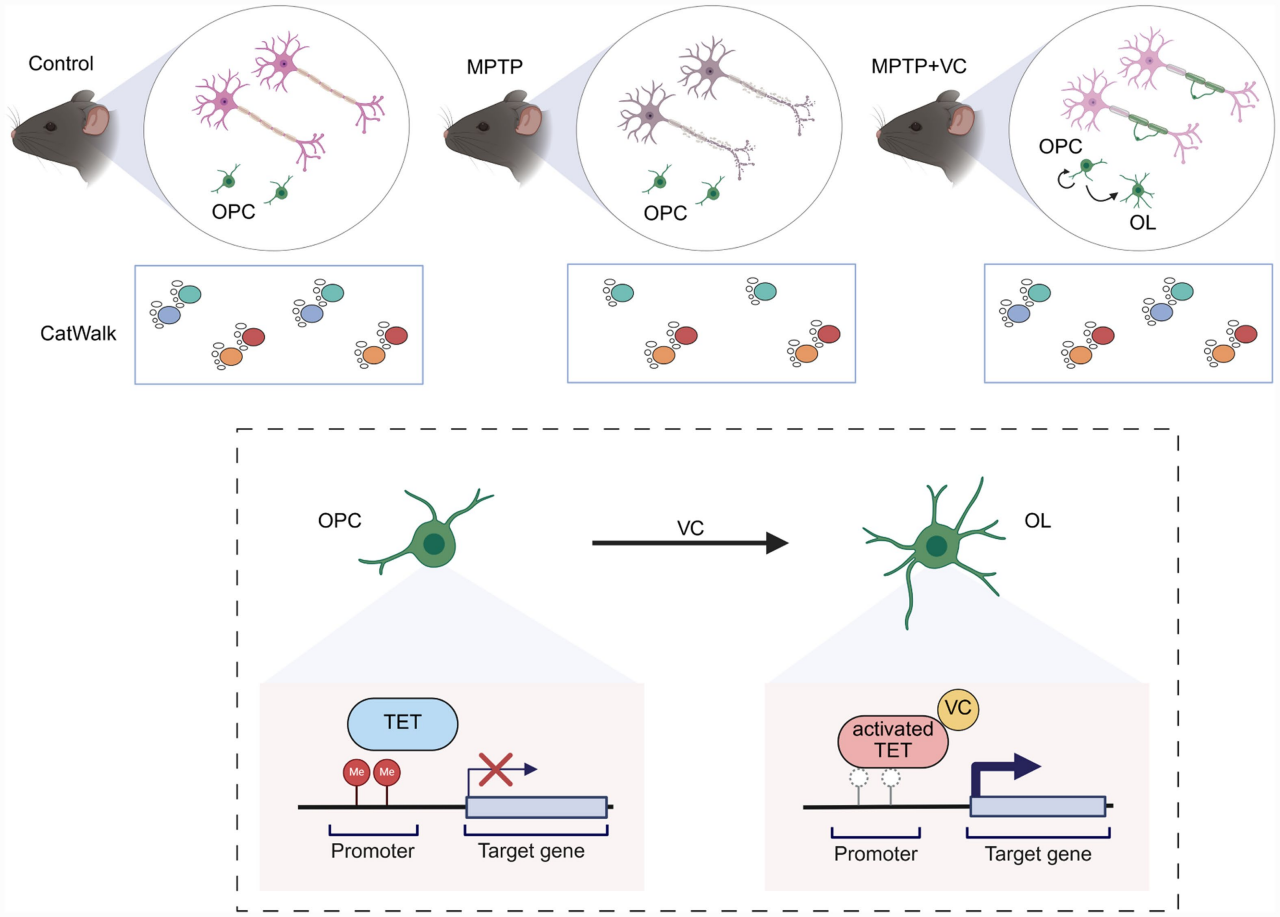
Schematic diagram of the mechanism for VC-mediated enhancement of oligodendrocyte generation and myelin renewal in a PD model. The schematic illustrates the therapeutic effects of VC in a PD model. Top: The brain showing neuronal demyelination and degeneration, accompanied by impaired gait patterns in MPTP-induced chronic PD mouse model. VC administration promotes the differentiation of OPCs into myelinating OLs, leading to myelin repair and amelioration of motor deficits. Bottom: VC activates TET enzymes, promoting DNA demethylation at target gene promoters and enabling transcription, thereby driving OPC to OL differentiation and myelin renewal. Created in BioRender.

Based on the findings above, we proposed a schematic model for VC-mediated neuroprotection through enhanced OPC-to-oligodendrocyte differentiation in a murine model of PD (Figure 6). In control mice, oligodendrocytes wrap dopaminergic neuron axons to maintain myelin integrity, supporting proper dopaminergic neuron function and normal gait patterns. MPTP exposure impairs dopaminergic neuron, leading to impaired OPC differentiation, reduced OL numbers, myelin damage, and associated motor deficits characterized by irregular gait patterns. Our findings indicate that VC administration reverses these deficits by promoting OPC-to-OL differentiation, thereby facilitating myelin renewal and restoring motor function. At the mechanism level, VC acts as a cofactor to activate TET enzymes, which catalyze the demethylation of target gene promoters that are critical for OPC-to-oligodendrocyte differentiation, likely facilitating the transcriptional activation of OL differentiation programs. The enhanced oligodendrogenesis promotes myelin renewal, protects dopaminergic neurons, and ultimately ameliorates motor and cognitive deficits in the MPTP-induced PD model. These findings establish VC as a potential therapeutic agent that targets oligodendrocyte dysfunction and myelin pathology in Parkinson’s disease.

## 4 Discussion

Severe motor dysfunction is a defining clinical hallmark of PD, primarily attributed to the progressive degeneration of nigrostriatal dopaminergic neurons. While neuronal pathology has been the central focus of PD research, growing clinical and preclinical evidence implicates significant white matter abnormalities and OL dysfunction in disease pathogenesis (Yang et al., 2023; Barba-Reyes et al., 2025; Li et al., 2025; Zhang et al., 2025). Another study taking a whole brain connectomics approach also suggested a significantly decreased myelin content in PD patients (Boshkovski et al., 2022). In this study, we used scRNA-seq combined with WB to reveal significant OL and myelin loss in the brains of an MPTP-induced PD mouse model, consistent with the findings of OL pathology in human PD brains (Smajić et al., 2022; Barba-Reyes et al., 2025). Based on these findings, it is plausible that OL dysfunction is an integral component of PD pathology and myelin loss exacerbates dopaminergic neuronal degeneration and contribute to PD-related phenotypes. As observed in an Alzheimer’s disease (AD) mouse model where myelin deficits contribute to cognitive decline and enhancing myelin renewal can rescue cognitive function (Chen et al., 2021), we investigated the therapeutic potential of promoting myelination in PD. Here, our study demonstrates that VC acts as a potent pro-myelinating agent in a mouse model of PD. By enhancing OL generation and myelin renewal, VC treatment protects dopaminergic neurons and mitigates PD-related phenotypes.

The underlying mechanisms responsible for myelin and OL loss in PD are currently unclear. Our scRNA-seq analysis showed a notable enrichment of ferroptosis-related genes in OLs, which might be a crucial reason for the dysfunction and loss of OL in PD mice. A few studies reported the presence of α-synuclein inclusions in OLs from PD patient brains (Wakabayashi et al., 2000; Djelloul et al., 2015) and the accumulation of α-synuclein in OLs has been shown to cause demyelination (Poewe et al., 2022). In addition, brain-wide oxidative stress is an important feature of PD, and OL and OPC are known to be highly vulnerable to oxidative damage (Spaas et al., 2021). The death of OLs creates a vicious cycle, as the resulting demyelination not only impairs neuronal function but also triggers a neuroinflammatory response that can further damage both neurons and the remaining OLs. Following demyelination, OPCs are recruited to areas of injury, differentiate into OLs, which can be observed in demyelinating mouse models as well as in neurodegenerative disorders (Cayre et al., 2021). However, our results and a recent single-nucleus RNA sequencing of human PD brains have revealed compromised oligodendrocyte differentiation and a downregulation of key myelin-related genes. These results suggest that endogenous generation of OLs is often insufficient to fully compensate for extensive myelin loss in PD. Hence, our study demonstrated that VC robustly enhances the generation of OLs from their precursors, thereby increasing the pool of myelinating cells available for repair and significantly ameliorated myelin damage. More important, this was accompanied by the preservation of dopaminergic neurons and a marked improvement in behavioral performance. Our findings align with and extend previous work showing that pro-myelinating strategies can alleviate functional deficits in PD. For example, a recent study demonstrated that the overexpression of the transcription factor STAT5B specifically in oligodendrocytes could ameliorate myelin impairment and protect dopaminergic neurons in mouse models of PD (Li et al., 2025). Another study used the traditional Chinese medicine to reduce oligodendrocyte apoptosis and improved the movement disorders in MPTP-induced PD mice (Lei et al., 2025).

The therapeutic potential of VC in neurodegenerative diseases has historically been attributed to its potent antioxidant capacity (Covarrubias-Pinto et al., 2015; Ballaz and Rebec, 2019; Song et al., 2021), which helps neutralize the excessive reactive oxygen species (ROS) generated during neurodegeneration. While this mechanism likely contributes, our findings significantly expand the therapeutic profile of VC by revealing its role as a direct regulator of oligodendrocyte differentiation and myelin repair. Furthermore, chronic MPTP administration, as employed in our study, produces mild and prolonged neurodegeneration, with the nigrostriatal pathway remaining relatively preserved during early exposure phases (Ayerra et al., 2024). We therefore propose that oligodendrocyte damage occurs concurrently with, or may even precede, overt dopaminergic neuron loss in this model. The relationship between oligodendrocyte dysfunction and neuronal degeneration is likely bidirectional: myelin loss deprives axons of metabolic support and accelerates neurodegeneration, while neuronal dysfunction further compromises oligodendrocyte homeostasis, creating a self-amplifying pathological cycle. In this context, VC was administered throughout the entire MPTP treatment period, allowing for continuous action on oligodendrocyte lineage cells. By promoting OPC-to-OL differentiation, myelin renewal, and oligodendrocyte survival, VC likely mitigates dopaminergic neuron loss through: (i) restoration of myelin-mediated axonal metabolic support, (ii) enhanced secretion of oligodendrocyte-derived neurotrophic factors, and (iii) attenuation of oligodendrocyte-driven neuroinflammation. It is plausible that these functions act synergistically to produce the observed neuroprotective effects, suggesting VC could be an excellent agent against the complex pathology of PD. Furthermore, a study has shown that VC can reduce neuroinflammation by the modulation of microglial responses and astrocyte activation in a PD mouse model, thereby mitigating the loss of dopaminergic neuron (De Nuccio et al., 2021). In addition, VC is also believed to enhance the differentiation of dopaminergic neuron *in vitro* (He et al., 2015). Moreover, recent studies demonstrating that oligodendrocyte-derived GPR37 upregulation and PSAP secretion contribute to neuroinflammation and dopaminergic degeneration in MPTP models (Ma et al., 2025). These findings reinforce the concept that oligodendrocytes are not merely passive bystanders but active participants in PD pathophysiology, and that targeting oligodendrocyte dysfunction represents a valid therapeutic strategy.

An important anatomical consideration is that nigral dopaminergic neurons are largely unmyelinated or sparsely myelinated (Braak et al., 2004; Orimo et al., 2011). The sparse myelination of DA neurons contributes to their vulnerability, but this very vulnerability may render them particularly dependent on oligodendrocyte support functions. In this context, oligodendrocyte loss or dysfunction would disproportionately impact DA neurons that lack the protective buffering provided by robust myelination. First, oligodendrocytes serve as vital metabolic partners, supplying lactate and pyruvate to axons to support their high energy demands (Lee et al., 2012). Furthermore, oligodendrocytes secrete essential neurotrophic factors, such as GDNF and BDNF, which are required for dopaminergic neuron survival (Wilkins et al., 2003). Our immunofluorescence data demonstrating reduced GST-pi^+^ OLs number in both the SN and striatum of MPTP-treated mice, with restoration by VC treatment (Figures 4H–J), suggest that VC may preserve oligodendrocyte populations and their supportive functions may provide greater benefit to these vulnerable neurons. Finally, while proximal nigrostriatal axons are unmyelinated, distal projections exhibit partial myelination, and the substantia nigra receives extensive afferent inputs from cortical, thalamic, and other subcortical regions, many of which are myelinated (McGregor and Nelson, 2019). Following MPTP-induced injury, there may be increased demand for remyelination of demyelinated or dysmyelinated axonal segments. Taken together, our data show that VC promotes OPC-to-oligodendrocyte differentiation and increases mature oligodendrocyte numbers, potentially enhancing endogenous remyelination capacity and accelerating functional recovery.

VC is a well-known co-factor for the TET family of DNA dioxygenases, which catalyze the conversion of 5mC to 5hmC (Minor et al., 2013). This 5hmC modification is a key intermediate in active DNA demethylation and is particularly abundant in the brain, where it plays a vital role in regulating gene expression during OL development (Santiago et al., 2014; Zhao et al., 2014; Antunes et al., 2019). TET enzymes are crucial drivers of OL formation and myelin repair. Specifically, TET1-mediated DNA demethylation is indispensable for developmental myelination and efficient repair in the adult central nervous system. Its deficiency is known to impede myelin development and exacerbate age-related declines in remyelination (Moyon et al., 2021; Zhang et al., 2021). While TET2 and TET3 also contribute to OL differentiation, they are characterized by distinct spatiotemporal expression patterns, suggesting specialized roles during the process (Zhao et al., 2014). Our scRNA-sequencing analysis revealed the downregulation of Tet1-3 in PD mouse OLs, directly linking TET dysfunction to PD pathology. Our study provides evidence that this cofactor role of VC is directly relevant to OL differentiation. We found that VC treatment leads to a significant increase in global 5hmC levels in OPCs, and this increase is concomitant with the enhanced expression of OL marker MBP. When we blocked the formation of 5hmC using specific inhibitors, the ability of VC to promote OPC-to-OL differentiation was almost completely abolished. This finding aligns with recent evidence demonstrating TET’s necessity for OL differentiation (Ren et al., 2024) and extends these observations in stroke models where VC increased TET activity and 5hmC levels (Morris-Blanco et al., 2022) to the PD context. Based on the above results, we speculate that VC, by enhancing TET activity, facilitates the demethylation and subsequent transcriptional activation of genes critical for OL maturation and myelination, such as Olig2, Sox10, and MBP. Of course, our mechanism studies focus on global TET activity. The precise genome-wide methylation changes and target genes mediating VC’s effects remain to be fully elucidated.

There are several translational considerations and key areas warrant further investigation in future studies. First, regarding pharmacokinetics and dosing feasibility, the intraperitoneal route of VC administration used in this study is appropriate for mechanistic investigations in rodent models but is not directly applicable to clinical settings. In humans, oral VC produces plasma concentrations that are tightly controlled at approximately 70–85 μM even at high oral doses (Levine et al., 1996, 2001). In contrast, intravenous (IV) administration can achieve millimolar plasma concentrations (Padayatty et al., 2004), which may be necessary to elicit pharmacological effects. Notably, high-dose IV VC has been safely administered in oncology clinical trials, providing a potential framework for neurological applications (Parrow et al., 2013).

Second, optimizing the delivery of vitamin C to the central nervous system represents a primary focus for future translational efforts. VC transport across the blood–brain barrier (BBB) is primarily mediated by sodium-dependent vitamin C transporter 2 (SVCT2) expressed on choroid plexus epithelium (Harrison and May, 2009), which may limit CNS bioavailability under standard dosing regimens. In our study, we employed ascorbic acid 2-phosphate (As-2P), a stable and oxidation-resistant form of VC. However, additional strategies could further enhance therapeutic efficacy, including nanoparticle-based delivery systems that facilitate BBB penetration, liposomal formulations with prolonged circulation time, or lipophilic prodrugs of VC that can more effectively cross the BBB through passive diffusion.

Third, another important direction is to explore combination therapies. Given that the intrinsic capacity for remyelination can be limited by age and disease, combining VC with other pro-myelinating agents or inhibitors of pathways that block OL differentiation, such as Wnt or Notch pathways, could yield synergistic benefits. For example, several agents with dual neuroprotective and remyelinating properties show promise for PD. Fasudil, a ROCK inhibitor currently in clinical trials for PD (Wolff et al., 2024), has demonstrated these effects in multiple sclerosis (MS) (Wang et al., 2022; Wolff et al., 2024). Similarly, Catalpol has exhibited neuroprotective efficacy in the MPTP mouse model of PD (Wang et al., 2019) and exerted the ability to promote oligodendrocyte regeneration and remyelination via regulation of the NOTCH1 signaling pathway (Sun et al., 2021). Fingolimod, an FDA-approved MS drug, also exerts therapeutic effects in PD mouse models by enhancing oligodendrocyte survival and function (Zhao et al., 2017; Rajan et al., 2024). Such combinatorial approaches may produce synergistic effects on oligodendrocyte survival, OPC-to-oligodendrocyte differentiation, and myelin repair, potentially allowing for lower individual drug doses while maintaining or enhancing therapeutic outcomes.

Several important limitations must be acknowledged. First, a limitation of our scRNA-seq approach is that whole-brain tissue was used for cell isolation, which precludes direct assessment of region-specific transcriptional changes within the oligodendrocyte lineage. Future studies employing spatial transcriptomics or region-specific single-cell isolation would further resolve the regional heterogeneity of oligodendrocyte responses in PD models.

Second, it should be noted that DMOG, the pharmacological inhibitor used to block TET activity, is not cell-type specific and may affect TET-mediated processes in neurons and other glial populations in addition to OPCs/oligodendrocytes. Therefore, our mechanistic conclusions regarding the TET-mediated DNA hydroxymethylation in VC-induced OPC-to-oligodendrocyte differentiation are primarily supported by *in vitro* experiments using isolated OPC cultures, where we directly demonstrated that VC enhances OPC-to-oligodendrocyte differentiation and that this effect is blocked by DMOG. Future studies employing conditional, cell-type-specific genetic approaches, such as Cre-loxP-mediated deletion of Tet1/2/3 in the oligodendrocyte lineage will be necessary to definitively establish the cell-autonomous role of TET enzymes in VC-mediated oligodendrogenesis and remyelination *in vivo*.

In conclusion, our findings establish VC as a promising therapeutic candidate for PD and strengthen the rationale for targeting OL-mediated myelin repair to halt or slow disease progression. By acting as a cofactor for TET enzymes and promoting DNA hydroxymethylation, VC can effectively enhance the generation of OL and myelin renewal, leading to the protection of vulnerable dopaminergic neurons and corresponding improvements in behavioral deficits.

## Data Availability

The data that support the findings of this study are available from the corresponding author upon reasonable request.
